# Usefulness of empiric superior vena cava isolation in paroxysmal atrial fibrillation ablation: a meta-analysis of randomized clinical trials

**DOI:** 10.1007/s10840-024-01867-y

**Published:** 2024-08-09

**Authors:** Marco Valerio Mariani, Marta Palombi, Jean Pierre Jabbour, Nicola Pierucci, Pietro Cipollone, Agostino Piro, Cristina Chimenti, Fabio Miraldi, Carmine Dario Vizza, Carlo Lavalle

**Affiliations:** 1 Department of Cardiovascular, Respiratory, Nephrological, Aenesthesiological and Geriatric Sciences “Sapienza” University of Rome, Rome, Italy; 2 Cardio Thoracic-Vascular and Organ Transplantation Surgery Department, Policlinico Umberto I Hospital, Rome, Italy

**Keywords:** Atrial fibrillation, Catheter ablation, Pulmonary vein isolation, Non-pulmonary vein foci, Superior vena cava

## Abstract

**Background:**

The long-term success rate of pulmonary vein isolation (PVI) is suboptimal due to the presence of non-pulmonary vein (PV) foci that can trigger atrial fibrillation (AF) in up to 11%. Among non-PV triggers, the superior vena cava (SVC) is a major site of origin of ectopic beats initiating AF.

**Objective:**

To compare data from randomized controlled trials (RCTs) assessing PVI + empiric SVC isolation (SVCI) versus PVI alone in terms of AF recurrence, procedure-related complications, and fluoroscopic and procedural times.

**Methods:**

A search of online scientific libraries (from inception to April 1, 2024) was performed. Four RCTs were considered eligible for the meta-analysis totaling 600 patients of whom 287 receiving PVI + SVCI and 313 receiving PVI alone.

**Results:**

In the overall population, SVCI + PVI was associated with a non-significant reduction of AF recurrence at follow-up (0.66 [0.43;1.00], *p* = 0.05, *I*^2^ 0%). In patients with paroxysmal AF (PAF), a significant reduction of AF recurrence was related to SVCI + PVI (11.7%) as compared to PVI alone (19.9%) (0.54 [0.32;0.92], *p* = 0.02, *I*^2^ 0%). No statistical differences were found among the groups in terms of fluoroscopic (3.31 [− 0.8;7.41], *p* = 0.11, *I*^2^ = 91%), procedural times (5.69 [− 9.78;21.16], *p* = 0.47, *I*^2^ = 81%), and complications (1.06 [0.33;3.44], *p* = 0.92, *I*^2^ = 0%).

**Conclusion:**

The addition of SVCI to PVI in patients in PAF is associated with a significant lower rate of AF recurrence at follow-up, without increasing complication rates and procedural and fluoroscopy times.

**Graphical Abstract:**

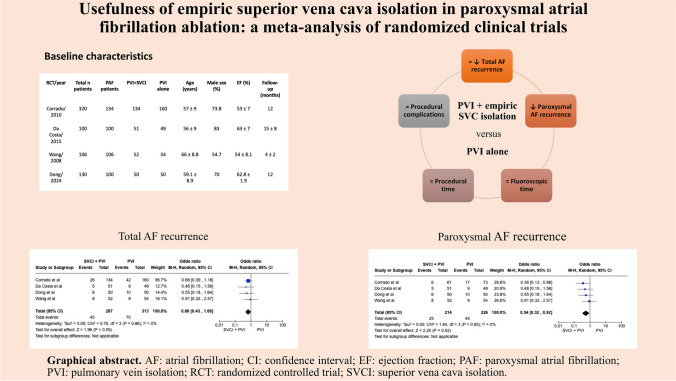

**Supplementary Information:**

The online version contains supplementary material available at 10.1007/s10840-024-01867-y.

## Introduction

Atrial fibrillation (AF) is the most frequently diagnosed arrhythmia in clinical practice [1]; it is associated with consistent morbidity and mortality, mostly due to heart failure (HF) and thromboembolic events, such as ischemic stroke and transient ischemic attack (TIA) [2]. Current evidence is directed toward rhythm control rather than rate control strategy, demonstrating to be beneficial in improving symptoms and the quality of life (QoL). Furthermore, a significant reduction of ischemic events and cardiovascular mortality was observed in the latest trials in patients who adopted rhythm control strategy as compared to rate control strategy [3, 4]. In this context, several studies showed that AF catheter ablation is more effective than antiarrhythmic pharmacological therapy in maintaining sinus rhythm and improving symptoms, either as first-line therapy or after antiarrhythmic drug (AAD) failure or intolerance [5–9].

Currently, pulmonary vein isolation (PVI) is the mainstay in AF catheter ablation, since pulmonary veins (PVs) represent the most important source of atrial ectopic beats initiating AF, originating in and around the PVs [2, 6, 7, 10–12]. However, besides AF recurrences related to PV reconnections during follow-up, the long-term success rate of AF ablation based on a PVI strategy is suboptimal due to the presence of non-PV foci that can trigger AF [13, 14]: superior vena cava (SVC), left atrium (LA) posterior wall, coronary sinus ostium, crista terminalis, interatrial septum, and ligament of Marshall. Among non-PV triggers, the SVC is a major site of origin of ectopic beats initiating AF, probably due to the arrhythmogenesis of SVC myocardial sleeves containing embryonic sinus venous tissue capable of increased automaticity and triggered activity [15]. Nevertheless, the usefulness of adding an empiric SVC isolation (SVCI) to PVI in reducing AF recurrences is unclear. In a non-randomized trial, Ejima et al. [16] demonstrated the superiority of an empiric SVCI strategy as compared to an as-needed approach in 186 paroxysmal AF (PAF) patients. Conversely, other non-randomized studies reported that the empiric SVCI in addition to PVI in redo AF ablation procedures did not reduce AF recurrences as compared to a PVI-only approach [17, 18]. Of note, these studies are quite different in terms of population and methodology. In the study by Knecht et al. [17], SVCI was performed only if < 2 PVs were reconnected at the time of redo procedure, whereas in the study by Simu et al. [18] SVCI was performed based on the discretion of the operator, with additional LA substrate ablation based on voltage map. Moreover, a high-power short-duration (HPSD) protocol was followed in latter study [18]. The methodological differences and the selected population (patients undergoing redo AF ablation procedure) make difficult the comparison among the studies and drawing conclusions on the effectiveness of empiric SVCI in addition to PVI.

To unravel this issue, we performed a meta-analysis of randomized clinical trials (RCTs) comparing PVI + empiric SVCI versus PVI alone in terms of AF recurrence, procedure-related complications, and fluoroscopic and procedural times.

## Methods

### Search strategy, selection criteria, and outcomes

The present meta-analysis was performed in accordance with the Preferred Reporting Items for Systematic Reviews and Meta-analysis (PRISMA) statement [19].

An online search of Pubmed, Web of Science, Cochrane Registry, Scopus, and EMBASE libraries (from inception to March 1, 2024) was performed, in addition to manual screening. We used the following keywords: [atrial fibrillation]; [catheter ablation]; [(pulmonary vein isolation) OR (pulmonary vein ablation)]; [superior vena cava] in various combinations. No language restriction was applied. We included RCTs comparing PVI + empiric SVCI versus PVI alone in the treatment of AF. Reviews, editorials, letters, meta-analysis, case reports, and abstracts were excluded.

The following outcomes were evaluated: AF recurrence in overall population and in the subgroup of patients with PAF, fluoroscopic and procedural times, and procedure-related complications (i.e., vascular access complications, thromboembolic events, cerebrovascular accidents, sinus node injury, pulmonary vein stenosis, phrenic nerve injury, and cardiac tamponade). AF recurrence was defined as any episode of AF longer than 30 s after the immediate post-ablation period, regardless of symptoms.

Two independent reviewers (MVM and MP) screened all abstracts and titles to identify potentially eligible studies, of which full text was subsequently interrogated. Agreement of the two reviewers was required for eligibility of studies for analysis. Disagreements regarding the inclusion or the classification of a study were solved by a third reviewer (JPJ).

### Data extraction and quality assessment

Data extraction was performed by two reviewers (MVM and MP). For each study the following data were collected: first author and year of publication, study design, population size, outcomes of interest (PAF recurrence, total AF recurrence, fluoroscopic time, procedural time, and procedure-related complications), antiarrhythmic therapy, ablative strategy (segmental ablation and circumferential ablation), months of follow-up, AF recurrence monitoring, and patients’ demographics (mean age, gender, types of AF, and mean left ventricular ejection fraction (LVEF)).

The quality of each study was assessed by evaluating specific elements of each study design using the Risk Bias Assessment Tool recommended by the Cochrane Collaboration [20].

### Statistical analysis

Descriptive analysis was based on counts (percentages) for dichotomous and categorical variables and as mean ± standard deviation (SD) for continuous variables or number of cases (*n*). Statistical analysis was performed using Review Manager (RevMan version 5.3, the Cochrane Collaboration, 2014; Oxford, UK). Statistical heterogeneity on each outcome of interest was quantified using *I*^2^ statistic and the Cochrane *Q* test. Values of *I*^2^ statistic ≤ 25%, 50%, and ≥ 75% indicated low, moderate, and high heterogeneity, respectively, whereas for *Q* statistic, substantial heterogeneity was defined as *p* < 0.1. Data were pooled using a random effect model, and the effect estimates chosen were the odd ratios (ORs) and weighted mean difference (WMD) with their corresponding 95% confidence interval (CIs), as needed. A sensitivity analysis was conducted using a fixed-effect model. A *p*-value < 0.05 was considered statistically significant.

## Results

### Study selection and patients’ characteristics

The literature search process identified 879 studies (Fig. A.1). After excluding duplicate publications, reviews, editorials, letters, meta-analysis, case reports, and abstracts, 17 studies were fully reviewed and 4 studies were considered eligible for the meta-analysis [21–24]; all the selected studies were RCTs. The overall population counted 600 patients (287 receiving PVI + SVCI and 313 receiving PVI alone); mean age was 58.6 ± 9.7 years, 71.7% were male. The mean follow-up went from 4.6 to 15 months, with the shortest follow-up duration in the study of Wang et al. [23]; the blanking period varied from 1 to 3 months. AADs were administered after the procedure in two studies [22, 23], whereas they were discontinued in the remaining two studies [21, 24]. Baseline characteristics of the included RCTs and study population are summarized in Table 1. Corrado et al. [21] included 320 patients, 134 with PAF and the last 186 with persistent and permanent AF. Wang et al. [23], Da Costa et al. [22], and Dong et al. [24] included only PAF patients (106, 100, and 100 patients, respectively). Overall, 440 PAF patients were included in the analysis. Remote magnetic navigation with 3D electroanatomical mapping system was used in the study by Da Costa [22] with an irrigated magnetic ablation catheter without contact force sensor. Wang et al. [23] and Dong et al. [24] used a 3D eletroanatomical mapping system, but Wang et al. [23] ablated with a 3.5-mm irrigated catheter without contact force capability, whereas Dong et al. [24] delivered radiofrequency with an irrigated catheter with contact force sensing. Corrado et al. [21] used non-irrigated catheters (an 8-mm tip catheter), without a 3D electroanatomical mapping system and without contact force sensor. Moreover, the definition of SVC-right atrium junction was achieved using different strategies in the studies: SVC angiography was used in the study by Wang et al. [23], intracardiac echocardiography (ICE) was used by Corrado et al. [21], and electroanatomical mapping with a multipolar catheter was used by Da Costa et al. [22] and by Dong et al. [24]. The quality of included studies was assessed using the Risk Bias Assessment Tool recommended by the Cochrane Collaboration, as shown in Table A.1.

**Table 1:** Baseline characteristics of the included randomized clinical trials (RCTs)

Study/year	Ablative strategy	Total n patients	PVI + SVCI	PVI alone	Inclusion criteria	Exclusion criteria		Primary outcome	AF recurrence monitoring	Blanking period (months)	Age (years)	Male sex (%)	EF (%)	AADs after ablation therapy
							Follow-up (months)							
Corrado [21]/2010	PVI + SVCI vs. PVI	320	134	160	Paroxysmal, persistent, permanent AF	LA thrombus, previous AF ablation	12	Success rate, outcome, complications of SVCI + PVI	ECG and Holter monitoring	2	57 ± 9	73.8	53 ± 7	no
Da Costa [22]	PVI + SVCI vs. PVI	100	51	49	Drug refractory, paroxysmal AF	Persistent and permanent AF, electrically silent RA-SVC junction, LA thrombus	15 ± 8	Recurrence rate of AF after ablation, freedom from atrial arrhythmias	Holter monitoring	2	56 ± 9	83	63 ± 7	At least 3 months
/2015														
Wang [23]	PVI + SVCI vs. PVI	106	52	54	Drug refractory, paroxysmal AF	LA thrombus	4 ± 2	Recurrence rate of AF after ablation	ECG and Holter monitoring	1	66.0 ± 8.8	54.7	54 ± 8.1	Amiodarone at least 1 month
/2008														
Dong [24]	SVC trigger: PVI + SVCI;	130 (SVC trigger: 30; non-SVC trigger: 100)	50	50	Drug refractory paroxysmal AF	Prior AF ablation, LA thrombus, severe structural heart disease	12	Freedom from atrial arrhythmias	Holter monitoring	3	59.1 ± 8.9	70	62.8 ± 1.9	no
/2024	no SVC trigger: randomization 1:1 PVI + SVCI vs. PVI alone													

*AAD*, antiarrhythmic drug; *AF*, atrial fibrillation; *ECG*, electrocardiogram; *EF*, ejection fraction; *LA*, left atrium; *PVI*, pulmonary vein isolation; *RA*, right atrium; *SVCI*, superior vena cava isolation

### Clinical outcomes

**Fig. 1:**
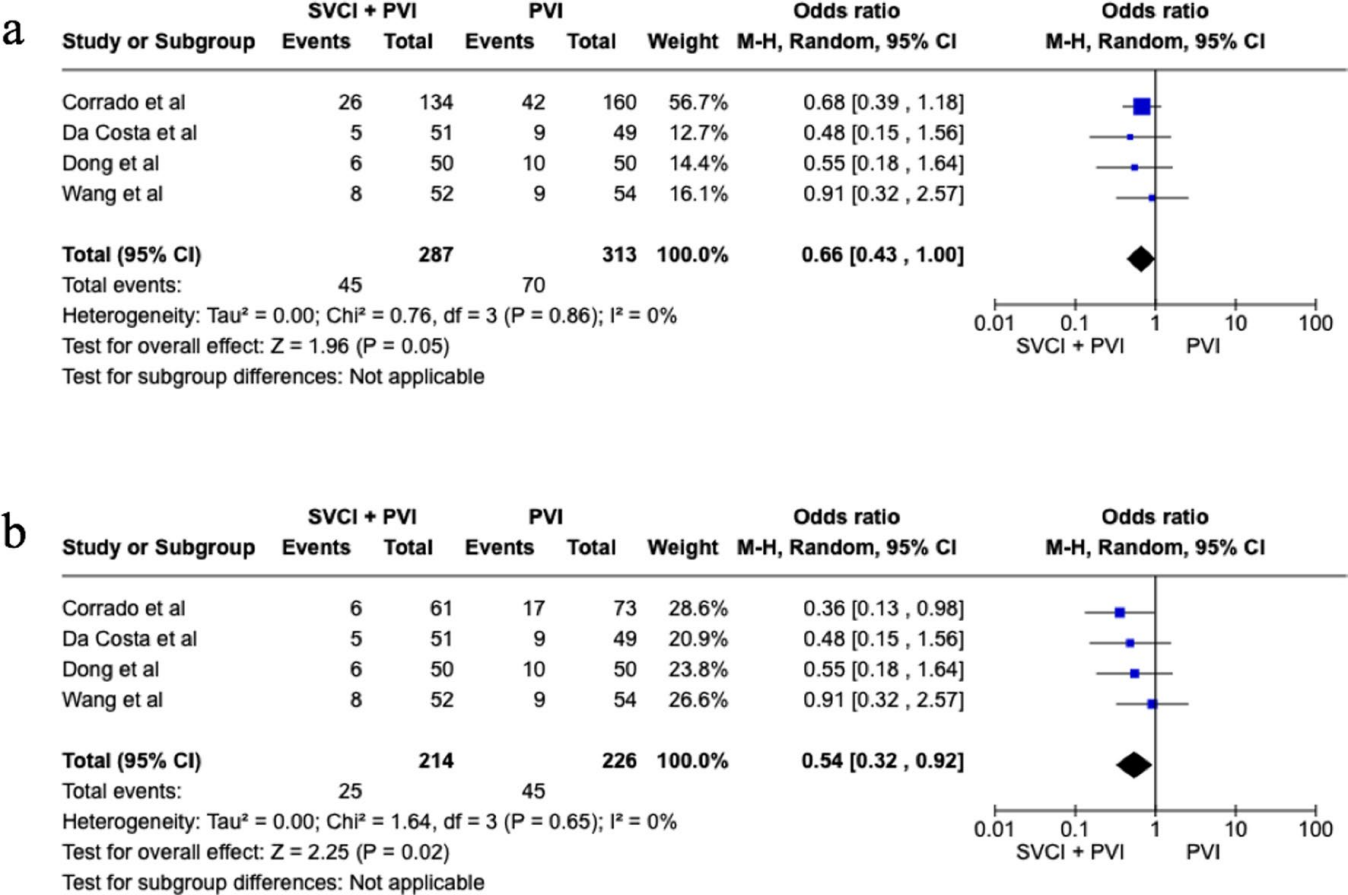
Forest plot of total atrial fibrillation recurrence (**a**) and paroxysmal atrial fibrillation recurrence (**b**). CI, confidence interval; PVI, pulmonary vein isolation; SVCI, superior vena cava isolation

**Fig. 2:**
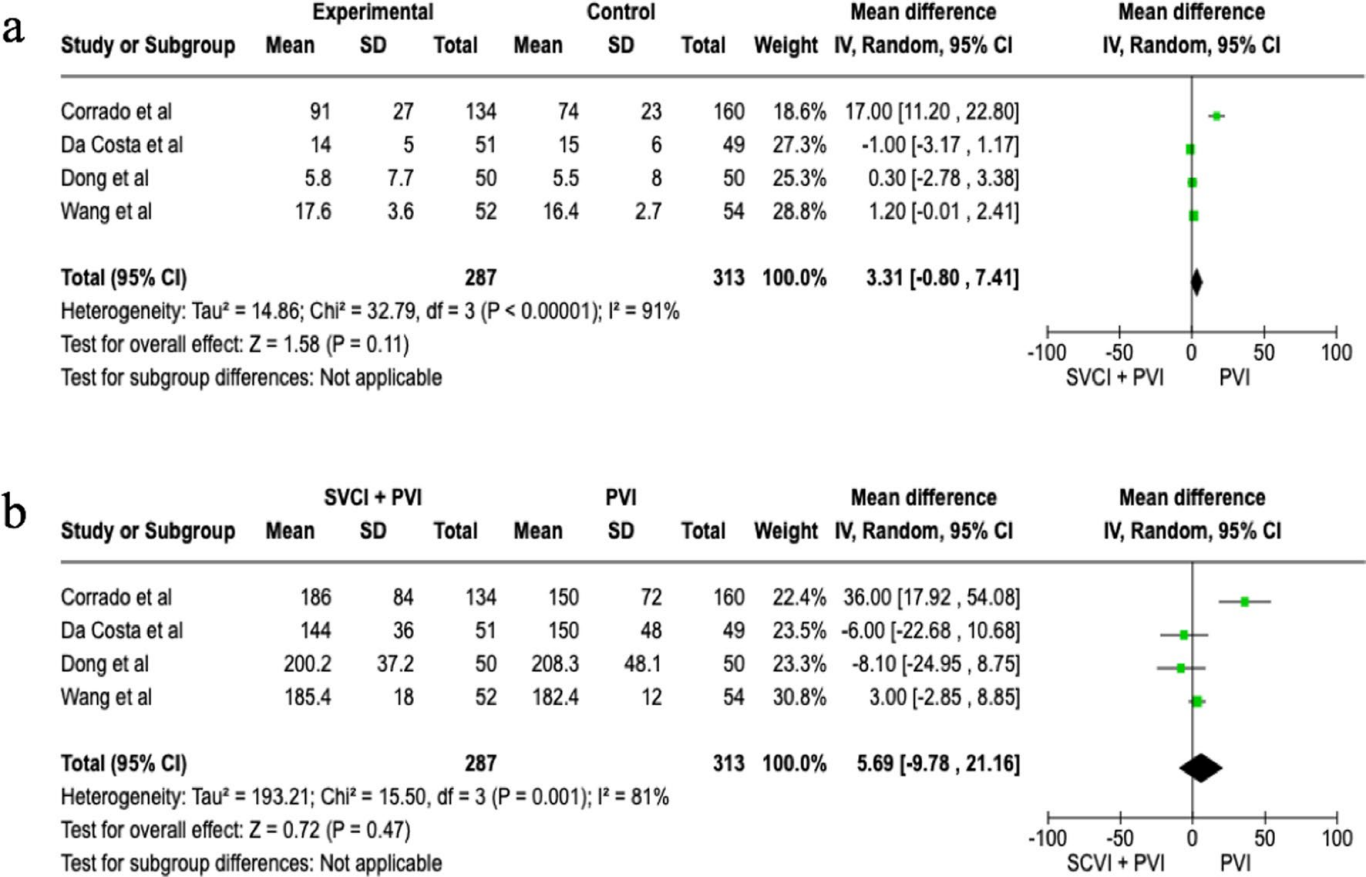
Forest plot of fluoroscopic time (**a**) and procedural time (**b**). CI, confidence interval; PVI, pulmonary vein isolation; SVCI, superior vena cava isolation

**Fig. 3:**
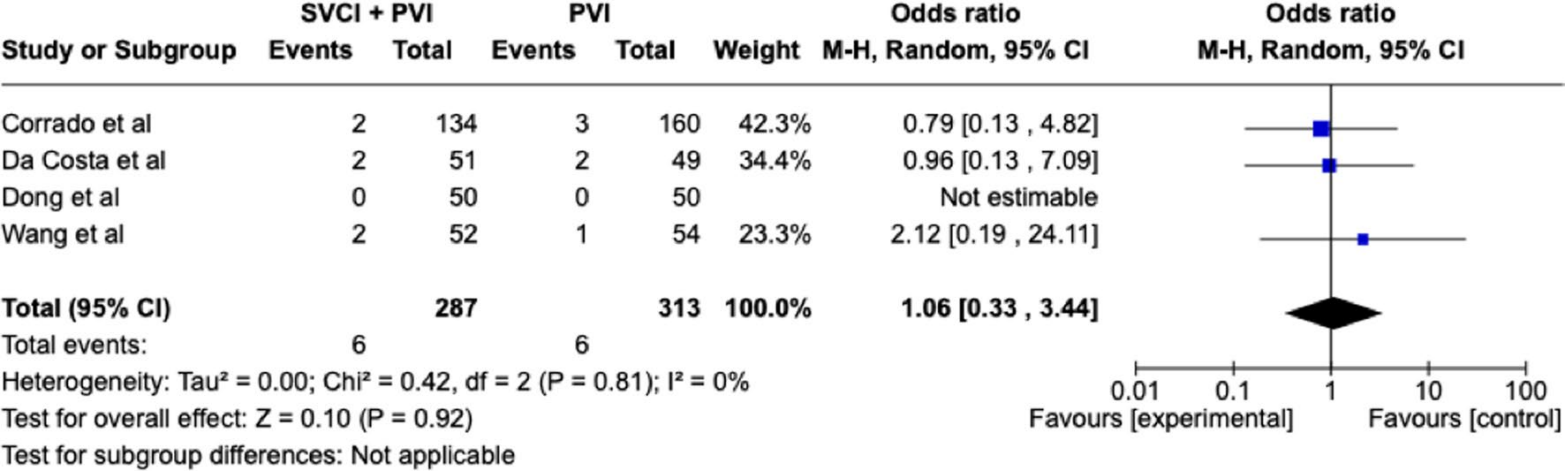
Forest plot of procedural complications. CI, confidence interval; PVI, pulmonary vein isolation; SVCI, superior vena cava isolation

In the overall population SVCI + PVI was associated with a non-significant reduction of AF recurrence at follow-up, although it showed a trend toward efficacy (OR 0.66 [95%CI 0.43;1.00], *p*-value 0.05, *I*^2^ 0%), whereas in patients with paroxysmal AF (PAF), a significant reduction of AF recurrence was related to SVCI + PVI (11.7%) as compared to PVI alone (19.9%), with a pooled OR of 0.54 ([95%CI 0.32;0.92], *p*-value 0.02, *I*^2^ 0%) (Fig. 1). No statistical differences were found among the groups in terms of fluoroscopic (WMD 3.31 [− 0.8;7.41], *p*-value 0.11, *I*^2^ = 91%) and procedural times (WMD 5.69 [− 9.78;21.16], *p* = 0.47, *I*^2^ = 81%) (Fig. 2). As for complications, there was no significant difference between SVCI + PVI versus PVI alone (OR 1.06 [0.33;3.44], *p* = 0.92, *I*^2^ = 0%) (Fig. 3). The sensitivity analysis using a fixed-effect model showed comparable results as compared with the random effect model analysis in terms of AF recurrence rates in the overall population and in the PAF population, as well as in terms of procedural time and complication rates (Figs. A.2–A.5). On the other hand, fluoroscopy time resulted significantly longer in patients who underwent SVCI + PVI as compared with PVI alone (WMD 1.11 [95% CI 0.12;2.10], *p*-value 0.03, *I*^2^ = 91%) (Fig. A.6).

## Discussion

Herein, we present the largest meta-analysis of RCTs exploring the effect of adding SVCI to PVI on AF recurrence in patients undergoing AF ablation. The main findings of the current meta-analysis are as follows:

- The addition of empiric SVCI to PVI in patients with PAF is associated with a significant 46% reduction in AF recurrence as compared with PVI alone;
- SVCI was not associated with a significant increase in terms of procedural and fluoroscopic times as related to PVI alone;
- SVCI did not result in an increased rate of peri-procedural complications compared with PVI alone strategy.

Embryologically, the SVC comes from a communication among the sinus venosus and the right atrium and contains cells with property of automaticity, due to the presence of phase 4 depolarization, and of triggered activity [25]. These cells are in myocardial extensions found inside the SVC, the so-called myocardial sleeves, which are responsible for arrhythmogenesis. In this view, it is not surprising that larger and longer myocardial sleeves are associated with higher arrhythmic properties, as recently confirmed by Dong et al. [24], who found that patients with SVC triggers had significantly longer SVC muscle sleeves as compared with patients without inducible SVC triggers. Of note, SVC is the most common site of origin of non-PV triggers, accounting for 25–40% of all non-PV triggers [24], and plays a role not only as a trigger but also as AF perpetuator. Indeed, in a series of 74 patients with SVC-associated AF, SVC initiated AF in 78.4% of cases, and in 32.4% of patients, SVCI was associated with AF termination, conversion to atrial flutter or persistence of atrial arrhythmias confined to the SVC, suggesting its role as perpetuator [26].

The involvement of SVC in AF strongly depends on AF patterns. As shown by Miyazaki et al. [26], an arrhythmogenic SVC was more prevalent between patients with PAF (8.5%), whereas in persistent and long-standing persistent AF, the prevalence was less than 2%. These findings suggest that SVCI may be especially beneficial in PAF patients and may explain the benefit of SVCI on AF recurrences found by Corrado et al. [21], who randomized 320 patients with paroxysmal, persistent, or permanent AF to receive PVI alone versus SVCI plus PVI. Although there was no difference in AF recurrence rates among the two-treatment groups at 1-year follow-up in the overall population, PAF patients undergone SVCI plus PVI had significantly less AF recurrences than PAF patients randomized to PVI alone [21].

The systematic review and meta-analysis from Sharma et al. [27] comprising the RCTs of Corrado et al. [21], Da Costa et al. [22], and Wang et al. [23] has previously shown a trend toward statistical significance in terms of reduction of AF recurrence solely in the PAF population, while no difference was found in total AF population when comparing SVCI + PVI with PVI alone. Conversely, our meta-analysis including the recent study of Dong et al. [24] showed a significant reduction in AF recurrence risk in PAF population, with a pooled OR of 0.54 ([95%CI 0.32;0.92], *p*-value 0.02, *I*^2^ 0%), and a trend toward an AF recurrence reduction in the overall population as well (OR 0.66 [95%CI 0.43;1.00], *p*-value 0.05,* I*^*2*^ 0%). Our meta-analysis reveals for the first time that the addition of empiric SVCI to PVI in patients with PAF is associated with a significant 46% reduction in AF recurrence as compared to PVI alone. The significant reduction in AF recurrences with SVCI found in the pooled analysis totaling 440 PAF patients suggests that the results of previous studies might have been hampered by their small sample sizes. In particular, the study by Wang et al. may have been also influenced by the short follow-up period and by the high incidence of PV reconnections among patients with AF recurrences in the SVCI plus PVI group, yet none of them demonstrated SVCI reconnections [23].

In patients with SVC trigger-induced AF, SVCI has demonstrated an effective ablation strategy. Chang et al. [28] achieved a 73% freedom-from-AF rate at 5-year follow-up after a single SVCI procedure and without PVI in patients with SVC-triggered AF. More recently, Dong et al. [24] found a 93.3% of AF freedom rate at 1-year follow-up in PAF patients with SVC triggers undergone SVCI plus PVI, showing the importance of SVCI in patients with inducible SVC triggers. However, they did not find significant benefit of adding empiric SVCI to PVI in patients without inducible SVC triggers (log-rank *p*-value 0.28), pointing against an empiric SVCI approach in PAF patients. The last three studies included in our pooled analysis enrolled unselected AF patients, with non-reported or a low rate of provoked SVC triggers elicited with a standard protocol, ranging from 3.1 to 3.7% [21, 23]. As a result, the meta-analysis includes a population without or with a very low rate of SVC-triggers. This consideration emphasizes our report of a significant 46% risk reduction of AF recurrence when empiric SVCI is added to PVI in a population mostly unselected for SVC triggers. Conversely to our results, the study by Dong et al. [24] showed that SVCI was not beneficial in patients without SVC triggers. The study by Dong et al. [24] used an aggressive protocol to elicit SVC triggers, with isoproterenol infusion, rapid burst pacing, and high doses of adenosine, that allows the recognition of higher rates of non-PV triggers (23.1% of patients showed SVC triggers). However, this induction protocol is time consuming and may be difficult to apply in daily clinical practice. Moreover, due to the transient nature of non-PV foci, several non-PV triggers may be unidentified despite aggressive provocative maneuvers, as shown by Miyazaki et al. [26]. In this view, an empiric SVCI approach may be preferred over an as-needed SVCI strategy and may explain our finding of significant AF recurrence reduction with SVCI in patients without or with a low rate of SVC-trigger inducibility.

Importantly, our meta-analysis showed that SVCI is not only effective but also safe. Indeed, no difference in terms of complications rates was found among the two groups (Table A.2), with two phrenic nerve injuries (PNI) occurring in the study by Da Costa et al. [22]. The incidence of PNI is low (0–5%), usually transient, and may be avoided searching phrenic nerve capture before ablation by pacing using high output. Moreover, the right phrenic nerve may be visualized by ICE during AF ablation, thus preventing its injury during radiofrequency delivery [29]. Yamaji et al. [30] investigated the optimal prevention method of PNI during SVCI and found that HPSD radiofrequency energy application (50 W, 7 s), only on SVC points where pacing stimulated the phrenic nerve, never resulted in PNI. Therefore, HPSD energy delivery may represent an optimal PNI prevention maneuver, due to the shallower and wider lesions as compared to the standard radiofrequency ablation. No sinus node injury has been reported in the meta-analysis, although previous study reported this complication in 1.1% of cases [31]. As shown by Dong et al. [24], electroanatomical mapping-guided SVCI allows the localization of the sinus node with successful ablation without any sinus node damage.

Overall, the current meta-analysis shows that the addition of SVCI to PVI seems to provide lower rates of AF recurrences at follow-up as compared with PVI alone, without increasing complication risk. However, our results must be interpreted with caution. Indeed, the number of complications was too limited to draw solid conclusions about safety of SVCI. Furthermore, though we found a significant reduction in terms of AF recurrences in PAF patients, the small number of included patients and the heterogeneity in ablation strategies found among studies entail confirmation of our results in further larger, well-designed RCTs to fully address the effectiveness and safety profile of SVCI.

## Limitations

Our meta-analysis has several limitations. To begin with, we included four studies with small sample size, characterized by the use of different ablation and mapping techniques, as the studies were conducted at different times. Secondly, the included studies adopted different blanking periods, AAD therapy durations, and follow-up period. However, no statistical heterogeneity was found when analyzing the outcome AF recurrence. Moreover, AF recurrence rates may have been underestimated because asymptomatic episodes may be undetected when using ECG and Holter monitoring. However, this should underestimate recurrences in both treatment arms. Eventually, the number of complications was too small to draw conclusions on the safety profile of PVI + SVCI strategy as compared to PVI-alone strategy.

## Conclusions

The addition of SVCI to PVI in patients in PAF is associated with a significantly lower rate of AF recurrence at follow-up, with a low complication rate, and without increasing procedural and fluoroscopy times. Larger RCTs are needed to fully clarify the effectiveness and safety of adding empiric SVCI to PVI in reducing AF recurrence in PAF patients.

## Supplementary Information

Below is the link to the electronic supplementary material.

Supplementary file1 (DOCX 736 KB)
