# Supplementary material for: Usefulness of empiric superior vena cava isolation in paroxysmal atrial fibrillation ablation: a meta-analysis of randomized clinical trials
Source: J Interv Card Electrophysiol. 2024 Aug 9;68(1):93–100. doi: 10.1007/s10840-024-01867-y (PMC11832614; doi:10.1007/s10840-024-01867-y)
Supplement: Supplementary file 1 — Supplementary file1 (DOCX 736 KB) [file 10840_2024_1867_MOESM1_ESM.docx]

**Appendices**

**Figure A.1.** Study flow diagram.

**

**

**Figure A.2.** Forest plot of atrial fibrillation recurrence in overall population (fixed effect model). CI: confidence interval; PVI: pulmonary vein isolation; SVCI: superior vena cava isolation.

**
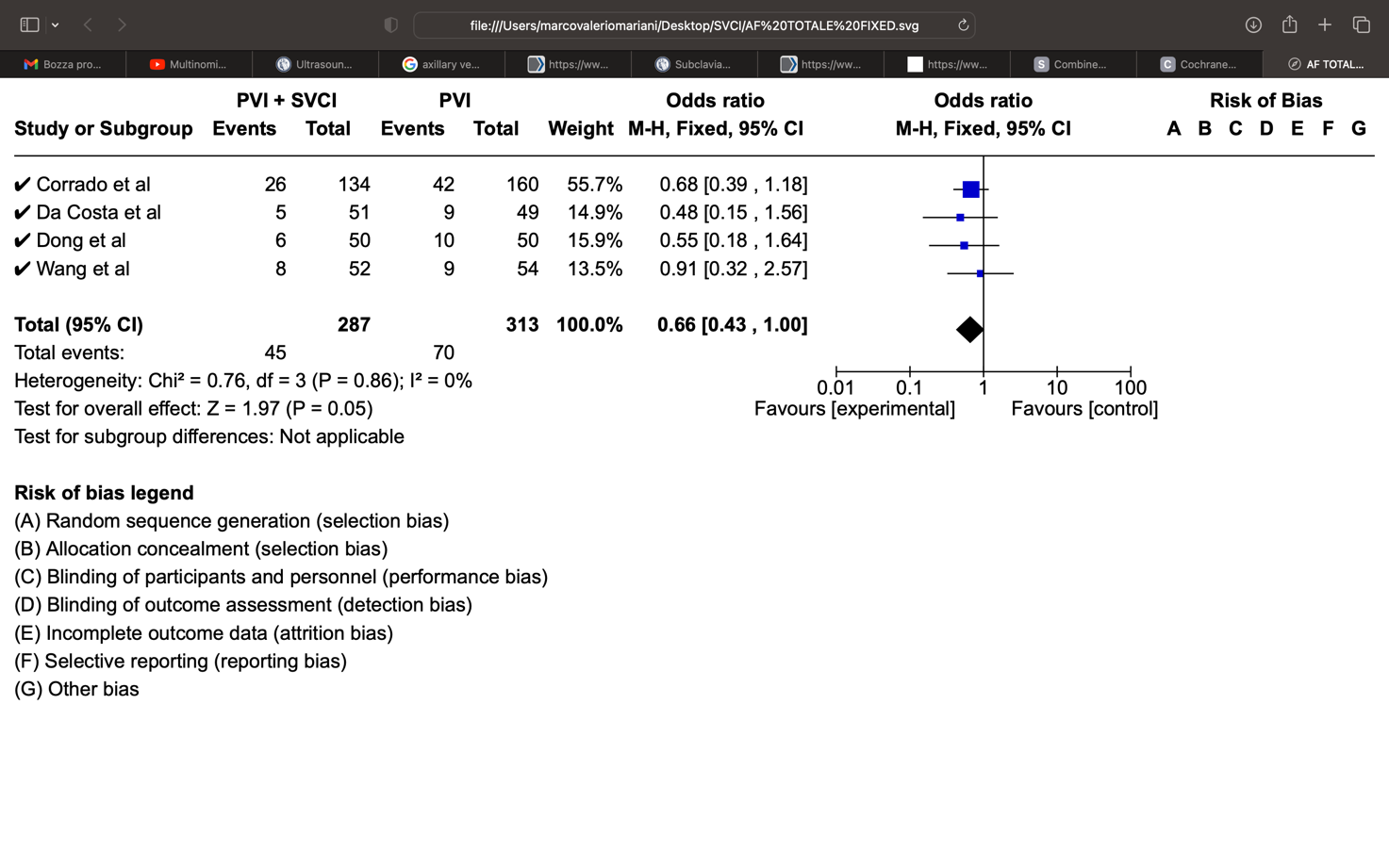
**

**Figure A.3.** Forest plot of atrial fibrillation recurrence in paroxysmal atrial fibrillation population (fixed effect model). CI: confidence interval; PVI: pulmonary vein isolation; SVCI: superior vena cava isolation.

**
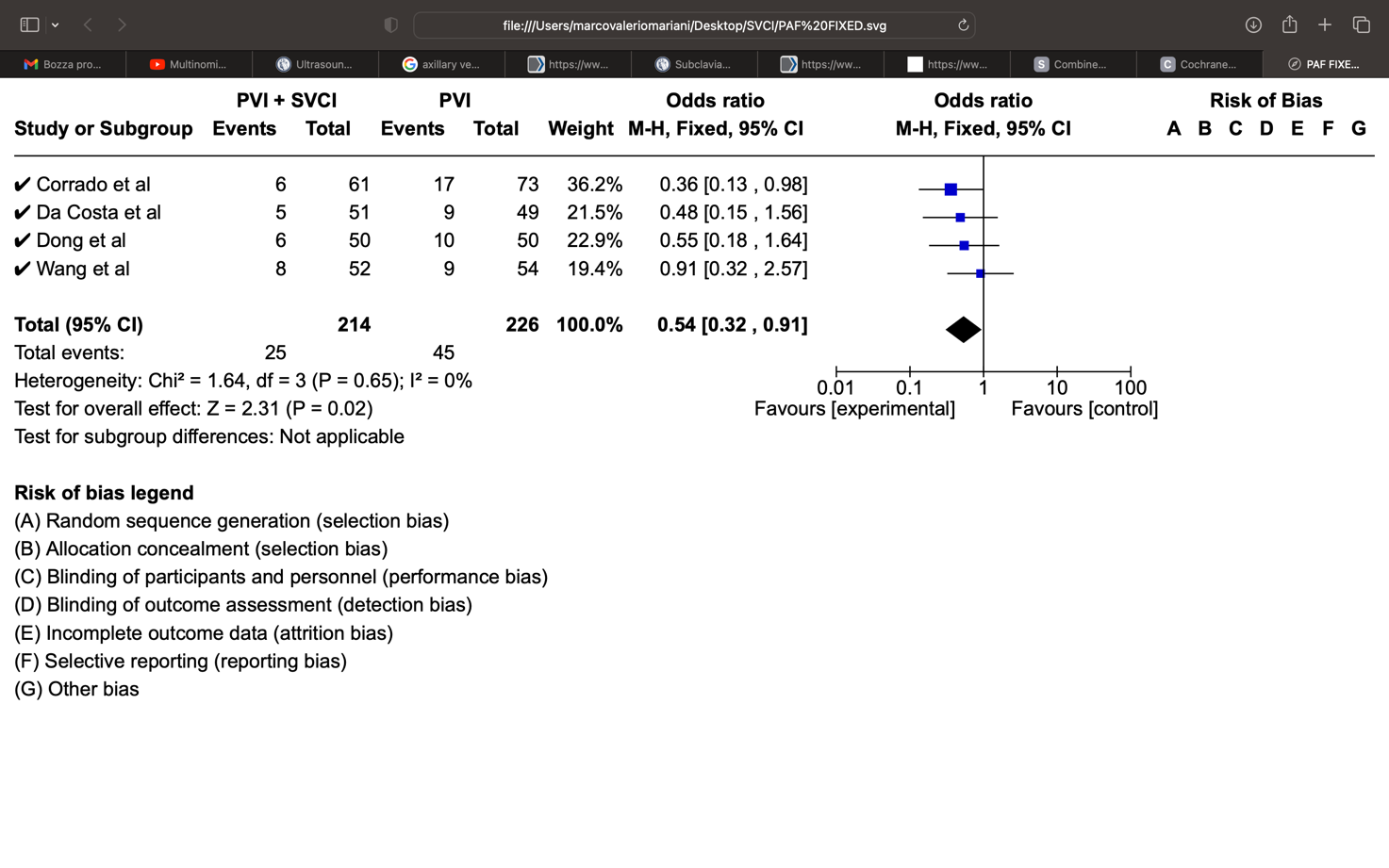
**

**Figure A.4.** Forest plot of complication rate (fixed effect). CI: confidence interval; PVI: pulmonary vein isolation; SVCI: superior vena cava isolation.

**
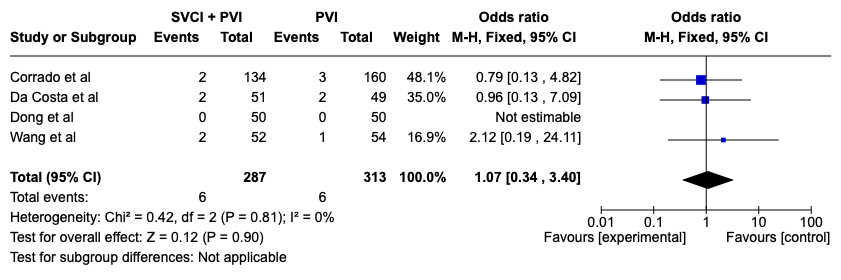
**

**Figure A.5.** Forest plot of procedural time (fixed effect). CI: confidence interval; PVI: pulmonary vein isolation; SVCI: superior vena cava isolation.

**
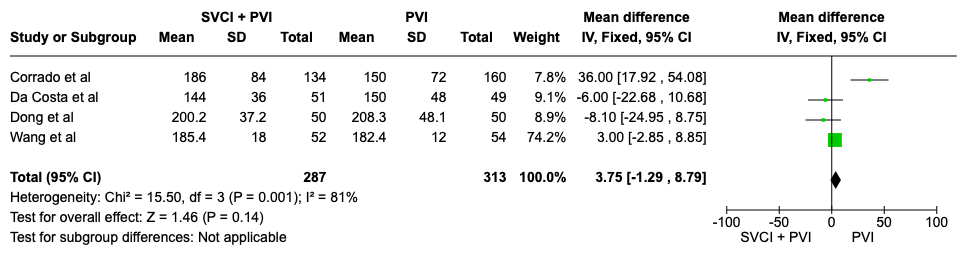
**

**Figure A.6.** Forest plot of fluoroscopic time (fixed effect model). CI: confidence interval; PVI: pulmonary vein isolation; SVCI: superior vena cava isolation.


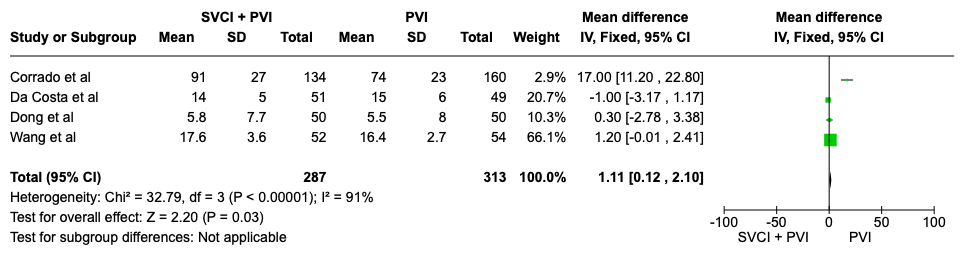


**Table A.1.** Risk of bias of individual studies by revised Cochrane Risk Assessment tool [19]

|  | Selection bias | Performance bias | Detection bias | Attrition bias | Reporting bias |
| --- | --- | --- | --- | --- | --- |
| Corrado ^(21)^ | + | +/- | +/- | +/- | + |
| Da Costa ^(22)^ | +/- | + | + | +/- | + |
| Wang ^(23)^ | + | + | + | +/- | + |
| Dong ^(24)^ | +/- | +/- | +/- | + | + |

Each field should be graded as: Low + (green) /High **–** (red) /Some concerns **+/-** (yellow).
